# Review of “*host manipulation by parasites* ” by David P. Hughes, Jacques Brodeur and Frédéric Thomas

**DOI:** 10.1186/1756-3305-5-228

**Published:** 2012-10-10

**Authors:** Hilary Hurd

**Affiliations:** 1 Institute for Science and Technology in Medicine, School of Life Sciences, Keele University, Keele, Staffordshire ST5 5BG, UK

## Review

**Hughes, D.P., Brodeur, J., Thomas, Thomas, F: ***Host Manipulation by Parasites.* Oxford University Press; 2012. 224 pages. ISBN 9780199642243

It is ten years since Janice Moore produced her comprehensive review detailing knowledge of parasites and animal behaviour, as it stood a decade ago. In the intervening time the field has moved on, and broadened its scope. This edited volume by Hughes, Brodeur and Thomas provides a timely and engaging synthesis of current information and opinions concerning the diverse ways in which parasites can manipulate their hosts to enhance their own fitness.

Each of the eleven chapters is followed by an aptly named afterword, almost all written by prominent experts on behavioural ecology rather than parasitology. These illuminating afterwords not only summarise and comment upon the preceding chapter but provide fresh insights into the particular topic. This useful and little used device adds much to the volume, making the intended link between these two disciplines. From the start, the style is accessible to novices to the field and Moore’s witty and entertaining history of the development of the field of host manipulation over the last three decades sets the scene for the eclectic nature of the remaining 10 chapters.

Most students of parasitology will have been introduced to the fascinating and bizarre stories of snails with striped, pulsating, tentacles full of *Leucochoridium paradoxum*, brain-worm-infected ants clamped by their jaws to the tips of vegetation and photophilic shrimps gyrating at the water’s surface as their acanthocephalan symbionts enhance their likelihood of being predated. Several contributors to this volume explore these favourite examples, but in addition, a refreshing variety of novel examples of host manipulation are discussed herein. I was particularly pleased to see Meschner’s insightful chapter on manipulation of plant phenotypes, intrigued that responses to herbivores were included and that discussions widened to consider these manipulations in the broader context of community and ecosystem levels; this later topic being expanded in a complete chapter on the ecological consequences of manipulative parasites. The inclusion of plants as hosts is an example of the expansive view the editors bring to this volume. Another unusual and thoughtful chapter on parasites of social organisms returns to the theme of the superorganism, discussed in Dawkin's foreword to the book, and considers the concept of a colony’s response to infection as well as that of an individual member. Likewise, the chapters on endosymbiotic bacteria as manipulators of insect reproduction, mimicry used by brood parasites to deceive their hosts and the agricultural and medical consequences of host manipulation, even as applied to human behaviour and personality, raise fascinating questions not usually addressed at meetings of parasitologists. Hughes, Brodeur and Thomas show us that the study of manipulative parasites is a topic that can break down conventional barriers between disciplines and expand into new fields.

The arrangement of the middle chapters appeared serendipitous but the second and final ones seem to act like bookends, the former viewing our limited understanding of the evolution of host manipulation and the latter expanding the theme and placing it in the context of manipulations that occur between the sexes, conspecifics and interspecies without the inclusion of a parasite phenotype.

Adamo poses the question “how do parasites manipulate their hosts?” and a few other authors touch on the mechanisms underlying manipulation of behaviour, but I was disappointed not to see much more on this topic, as, in my opinion, the answers will hold the key to understanding the evolutionary history of host manipulation. Perhaps this reflects our current state of knowledge.

The volume is aimed at graduate students and professional researchers in parasitology, evolutionary biology and behavioural ecology. The diverse nature of the material and approaches will undoubtedly provide something new and accessible for all of these readers. New entrants to the field will gain much from digesting the contents of this book; lecturers are likely to find new information, ideas and approaches to support their teaching and undergraduate students of parasitology may find some aspects challenging, but should be encouraged to dip into it.

Overall the topics covered indicate that host manipulation is moving into a new era beyond that of descriptive studies, but the book also shows this field may have developed past infancy, but still be at the toddler stage. If its participants continue to embrace the burgeoning fields of transcriptomics and proteomics or to collaborate with ecologists, who provide more expansive perspectives, or inject a mathematical approach (as illustrated in chapter 4), the toddler will flourish. This book could act as a call to arms for a new generation of parasitologists who are willing to embrace behavioural ecology. I recommend it highly.

## Competing interests

The author declares that she has no competing interests.

## Author’s contribution

HH was the sole author and approved the final manuscript.

